# “It’s really no more difficult than putting on fluoride varnish”: a qualitative exploration of dental professionals’ views of silver diamine fluoride for the management of carious lesions in children

**DOI:** 10.1186/s12903-020-01243-y

**Published:** 2020-09-15

**Authors:** Nassar Seifo, Heather Cassie, John Radford, Nicola Innes

**Affiliations:** 1 School of Dentistry, University of Dundee, Park Place, Dundee, DD1 4HR UK; 2 School of Dentistry, Cardiff University, Heath Park, Cardiff, CF14 4XY UK

**Keywords:** Silver diamine fluoride, Caries, Non-restorative caries treatment, Dental professionals, Barriers, Enablers

## Abstract

**Background:**

Despite evidence that Silver Diamine Fluoride (SDF) can be effective in managing carious lesions in primary teeth, the use of SDF in the UK remains limited. This study explored dental professionals’ views and experiences of using SDF for managing carious lesions in children. In addition, it explored what they perceived to be the advantages, disadvantages, barriers and enablers to the use of SDF in practice.

**Methods:**

Fifteen semi-structured face-to-face or over-the-phone interviews were conducted with 14 dental professionals from NHS Tayside and NHS Grampian in Scotland. Interviews were transcribed verbatim, coded and analysed using a thematic approach.

**Results:**

Thirteen of 14 dental professionals interviewed were familiar with, or had some existing knowledge of, SDF. Four had used it to treat patients. The majority of participants thought that the main advantage of SDF was that it required minimal patient cooperation. SDF was also perceived as a simple, pain-free and non-invasive treatment approach that could help acclimatise children to the dental environment. However, SDF-induced black staining of arrested carious lesions was most commonly reported as the main disadvantage and greatest barrier to using it in practice. Participants believed that this discolouration would concern some parents who may fear that the black appearance may instigate bullying at school and that others may judge parents as neglecting their child’s oral health. Participants thought that education of clinicians about SDF use and information sheets for parents would enhance the uptake of SDF in dental practice. Participants believed that younger children might not be as bothered by the discolouration as older ones and they anticipated greater acceptance of SDF for posterior primary teeth by both parents and children.

**Conclusion:**

Dental professionals were aware that SDF can be used for arresting carious lesions. They pointed out that the staining effect of carious lesions is a major disadvantage and had preconceived ideas that this could be a barrier for many parents. Participants considered the application process to be simple and non-invasive and requires a minimum level of child cooperation. Participants appreciated the potential of SDF in paediatric dentistry and suggested actions that could help overcome the barriers they highlighted.

## Background

Dental caries is the most prevalent chronic disease in the world [1]. In recent years, as a result of a better understanding of the pathology of the disease, there has been a shift from traditional ‘drill-and-fill’ techniques towards more minimal-intervention, evidence-supported treatment options, such as Atraumatic Restorative Treatment, the Hall Technique (HT) and fluoride agents [2]. The effectiveness of fluoride-based materials for preventing and arresting carious lesions is well-established [3, 4].

Silver nitrate was first reported as effective in arresting carious lesions in the 1840s [5] and G.V Black described protocols for its use in the early 1900s [6]. This paved the way for another silver product; silver diamine fluoride (SDF) to be developed. Silver diamine fluoride was first explored as a treatment option for managing carious lesions in Japan in 1969 [7]. It is a clear, odourless liquid [8] containing silver and fluoride, which act synergistically to arrest carious lesions through a variety of mechanisms [9]. Silver ions interact with the bacterial cell wall and enzymes to inhibit bacterial growth. They can also integrate into hydroxyapatite and have an antibacterial effect on silver-integrated hydroxyapatite. Furthermore, silver ions can inhibit cathepsins and dentine collagen degradation. On the other hand, fluoride can promote remineralisation by forming fluorohydroxyapatite with reduced solubility. It can also inhibit matrix metalloproteinases activities and therefore dentine collagen degradation [10].

Silver diamine fluoride was cleared by the Food and Drug Administration in the United States in 2014 for managing dentine hypersensitivity [11]. Since then, there has been growing interest in its “off-label” use for managing carious lesions, supported by reports of its effectiveness, with a recent umbrella finding it effective in arresting carious lesions in children [12].

There are two additional benefits of SDF as a treatment to arrest dental caries. Firstly, it is a non-aerosol generating procedure so can be applied in a way that does not increase the risk of transmission of acute respiratory infection (such as COVID-19 [13]) in the dental surgery. Secondly, it can be applied in settings other than the dental surgery because it does not require specialist dental equipment other than a mirror, tweezers and cotton wool.

It is well reported that the translation of evidence into practice is slow, unpredictable and can be met with resistance [14, 15]. The implementation of research findings requires more than simply producing and disseminating recommendations and clinical guidelines [16]. Despite its availability in the UK since April 2016 [17], use of SDF at the beginning of 2020 was still limited to dental schools and a small number of practices in the UK. Furthermore, there is scant research exploring Dental Professionals’ (DPs) views about using SDF to manage carious lesions in children [18–21]. Identifying the barriers and facilitators to its use in practice, from the perspective of DPs, may facilitate the development of strategies and interventions to improve its uptake.

This paper presents the findings from a qualitative study undertaken with DPs to explore their views, including their perceived barriers and enablers, to the use of SDF for the management of dental carious lesions in the primary dentition.

## Methods

### Study design

This study comprised semi-structured telephone and face-to-face audio recorded interviews with a purposive sample of DPs. Interviews took place between December 2018 and June 2019. The consolidated criteria for reporting qualitative research (COREQ) [22] were used as a guide to ensure quality.

### Participants and recruitment

To ensure sample diversity, a purposive strategy was adopted [23]. Dental Professionals who could apply topical fluoride or who were involved in its use, were considered eligible for inclusion. These included practicing dentists, dental therapists, dental hygienists and dental nurses, of any age. These clinicians were treated as a single group and no comparisons of views across professionals were made. Participants did not need to have previous experience of SDF application to take part.

Participants were recruited in two Scottish Health Boards (HBs) (NHS Grampian and NHS Tayside), through a Teaching Dental Hospital, general dental practices, Vocational Trainee Dental Practices, Public Dental Services and the Scottish Dental Practice Based Research Network’s database (SDPBRN) of Rapid Evaluation Practices.

Dental professionals were sent an invitation pack containing an information sheet, a reply slip and a freepost envelope. Participants were invited to return the reply slip in the freepost envelope or contact the researcher directly by telephone or email.

### Consent and ethical review

Prior to the interview, participants were given the opportunity to ask any questions and confirm they were happy to take part, and consent was explained and obtained. For face-to-face interviews, the consent process was carried out in person, while for over-the-phone interviews consent was discussed and then agreement to participate (if given) was audio recorded before the audio-recorded telephone.

This study was approved by University of Dundee Schools of Nursing, Health Sciences and Dentistry Research Ethics Committee (application number: 2018012_Seifo). The study was approved by the Research and Development Managements at NHS Tayside and NHS Grampian (IRAS ID: 252305).

### Data collection

Face-to-face to interviews took place at the workplace with the presence of the researcher and the participant only. Interviews were semi-structured using open-ended questions and probing. A topic guide was developed to explore the following: DPs existing knowledge and experience of SDF; perceived advantages and disadvantages to its use; barriers and enablers to SDF for the management of carious lesions in the primary dentition; and DPs views of children’s and parents/carers’ acceptability of SDF (Additional file 1: Interview topic guide).

The topic guide was piloted with two DPs and amended accordingly to ensure face validity. Data from pilot interviews were not included in the analysis. Interviews continued until data saturation was achieved, i.e. when no new themes or categories were emerging from the data. Interviews were carried out by one researcher NS who has experience of performing interviews as well as training in qualitative data management and analysis.

### Data handling and analysis

All audio recordings were securely transferred to a professional transcription service and transcribed verbatim. All identifiable data were anonymised. Transcripts were accuracy checked prior to analysis. Data were managed using NVivo 12 software QSR (International Pty Ltd., Melbourne, Australia). Thematic analysis was undertaken using the framework approach as a broad guide to organise and classify data according to key issues, concepts and emerging themes [24]. These interviews were exploratory, with the aim of identifying the advantages, disadvantages, barriers and enablers for using SDF in practice. It was therefore important that the method of analysis allowed for the identification of key issues using the topic guide as a broad framework as well as recognising other emergent themes.

To minimise bias and ensure consistency, a sample of transcripts were double coded independently and in duplicate by NS and HC (an experienced qualitative researcher).

A coding framework was developed following the initial review of three transcripts. This was then assessed by an independent researcher (HC), not involved in conducting the interviews. Development of the codebook was an iterative process with adaptations made through discussion as appropriate. The codebook is available on request.

### Patient involvement

Patients were not involved in this study.

## Results

Fifteen interviews were conducted (13 face-to-face and two by telephone) with 14 participants. One participant was interviewed twice, once prior to, and again after, applying SDF for the first time. No participants contacted refused to participate. Six participants worked within primary care and eight within secondary care. Of the 14 participants, 12 were dentists (nine general dental practitioners, one consultant, one core trainee and one vocational trainee), one dental therapist and one dental nurse. Nine of the14 participants were female. The age of participants ranged from 25 to 61 years with a clinical experience ranging from one to 33 years. Five participants had used SDF before. Most interviews lasted, on average 25 min in duration (ranging from 10 to 35 min), A few of the interviews were shorter when participants did not have much to discuss i.e. had not had considerable knowledge, or experience with SDF.

### DPs’ perceptions about using SDF in practice

The vast majority of those interviewed were aware of SDF and were able to articulate that it can be used for arresting carious lesions in children. A small number also identified that it can be used to treat dentine hypersensitivity or knew that it can be used as a topical fluoride to prevent caries. The black staining of arrested carious lesions was raised by most participants.

*“Um, so I know that it’s a method for arresting carious lesions, uh, and quite like with stainless steel crowns, it has a similar challenge, sometimes, to present to the parents that it’s not going to be very aesthetic because it’s going to stain them black”*

**DP 12 (Dentist)**.

Four participants reported that they had experience applying SDF before participating in the interviews although one was on extracted teeth only. One DP, a dental therapist, had not applied SDF at the time of their initial interview, however, had a patient booked in for SDF application. A second interview was conducted with this participant to gather feedback and explore their initial thoughts and experience. This made the total number of interviewed DPs with experience of SDF five. It was noted that that these five participants were all employees of Dundee Dental Hospital and School.

#### DPs’ perceived advantages of SDF

When participants were asked about the advantages of using SDF over other dental treatments, the majority highlighted that minimum cooperation was required; this could potentially be beneficial for children or patients with special needs or dental phobia. One dentist commented that this could result in reduced referrals to secondary care. It was however also highlighted that a degree of cooperation would still be required, given that SDF is prone to staining with anything it comes into contact.

*“I think it’ll be good for patients who we’ve got very little cooperation ……... So I think the children who have got developmental issues or erm, a low tolerance for dental treatment will be very good because there’ll be limited time where they’re in the chair”*

**DP 3 (Dental nurse)**.

The majority of DPs suggested that they believe SDF to be a simple, easy and non-invasive approach for managing carious lesions in children because there is no requirement for local anaesthetic, use of rotary instruments or even excavating carious tissues. One dentist who had used SDF several times commented:

*“I think it’s, it’s very easy, it’s very easy to do, it’s um, it doesn’t require us to do anything that a child will, will find particularly traumatic at all… Erm, it’s really no more difficult than putting on fluoride varnish”*

**DP 2 (Dentist)**.

Contrary to this, one dentist commented that not requiring an injection was not necessarily a unique advantage of SDF, highlighting other approaches used in children’s dentistry, such as HT.

Participants also suggested that due to being pain free and minimally invasive, SDF may help to acclimatise children to having dental treatment, helping them to be more aware of the dental environment and more accepting of more complex dental procedures in future visits. It may also help build a cooperative non dental-phobic patient through their adult life.

#### DPs perceived disadvantages and barriers of SDF

The majority of participants were concerned with the aesthetic outcomes of SDF treatment and suggested that the permanent discolouration of arrested carious lesions could potentially be a barrier to parents’ acceptability of its use. SDF can also stain the oral mucosa, skin and the clinic surface. Therefore, DPs highlighted that meticulous attention is required while applying it to avoid any inadvertent spillage or contact. Riva star™ (SDI Limited, Bayswater, Australia), which is the commercial SDF product available in the UK, is a clear solution. One dental therapist who reported having used this particular product reported that they found it inconvenient to use, because it was difficult to notice any accidental spillage before staining occurs.

*“Um, I would say the biggest disadvantage with something like silver diamine fluoride would be that get-getting patients to accept it, the fact that it might… they’ve maybe got lesions that are just pale brown or you know, not very highly coloured, when you paint this on it’ll actually turn them black so it’ll look quite unsightly”*

**DP 8 (Dentist)***.*

Participants believed that the aesthetics associated with SDF application would be the largest barrier from the parent’s and child’s perspective. There was an assumption, even from those who had not used SDF before, that parents may not agree to its use. Reasons given for this included, a fear of their child being bullied or a fear of judgment from others, who may think that they are not looking after their child’s teeth.

*“Um, I think there are some children where, um, if their teeth go dark chocolate brown they might get picked on at nursery or at school and, um, that certainly -- I have met children where that has been an issue”*

**DP 4 (Dentist)**.

Given the additional possibility of inadvertently staining the skin or the gingiva, one dentist suggested that parents may hesitate about choosing SDF unless they fully trusted the DP applying it. One participant mentioned having encountered patients reluctant to receive any fluoride treatment. They believed that SDF would not be an option for these patients:

*“There are some parents who believe fluoride is a poison and that is their belief and, um, despite the fact that you and I might think otherwise…”*

**DP 4 (Dentist)**.

Another disadvantage highlighted was the unpleasant taste or sensation attributed to SDF. In addition, participants with experience of applying SDF highlighted that it was not easy to access interdental lesions in posterior teeth unless the lesion was fully cavitated. In addition, food packing in that area might obstruct SDF from reaching the whole carious lesion. It was noted that the size of the micro-brush supplied with the SDF kit was not sufficiently small to access fully all interdental lesions in posterior teeth.

A number of participants suggested that the lack of training and information available about using SDF was a barrier to its use in general dental practice. However, this was less of an issue for DPs working within Dundee Dental School who reported that they had received training on its use.

*“I mean, obviously, I work in a teaching hospital so I get exposed to new techniques and things, but people in practice, unless they go on courses to learn how to use it, if they weren’t trained with it, they might be very reluctant to use it not knowing anything about it”*

**DP 1 (Dentist)**.

It was suggested that introducing a new fluoride agent into practice may be challenging due to another type of fluoride based preventive material, Fluoride Varnish (FV), having been used in practice for a significant period. In addition, since SDF is licenced for treating dentine hypersensitivity, using it to arrest carious lesions would be deemed “off-label”. Indeed, for this reason, some of those interviewed suggested they would be hesitant to use it, with one DP querying whether there could be legal implications using this “off-label”.

*“The off licence to me is more of an issue if you're trying to get it used in general practice because personally, I would feel less comfortable. Doesn’t mean I wouldn’t use it, it just means that I would be a bit more cautious in how I’d approach the children”*

**DP 2 (Dentist)**.

Dental Professionals working within NHS primary care highlighted an additional barrier with SDF not currently listed in the Scottish Statement of Dental Remunerations (SDR). As a result, practitioners in Scottish NHS primary care practices would not be able to claim financially for applying this agent.

#### DPs’ perceived enablers of SDF use

As well as capturing potential barriers to using SDF in practice, factors to enable its use were also explored. It was suggested that the lack of training opportunities available could be addressed with the development of new training courses or Continuous Professional Development events which may in turn encourage use in practice. In addition, educating DPs about the implications of using agents “off-label” would mitigate such concerns.

*“Um, so I think for me it was a barrier initially. Um, but then the more I read about it I realised that being used off licence is okay… Um, so I'm very happy to do it now”*

**DP 2 (Dentist)**.

It was suggested that in order to facilitate the introduction of SDF to parents, an information sheet, explaining the associated advantages, disadvantages and expected outcomes, with photographs demonstrating arrested carious lesions, could help.

*“It would be nice to have something official in place that they could read as well, that’s probably a good consideration”*

**DP 12 (Dentist)**.

**Fig. 1:**
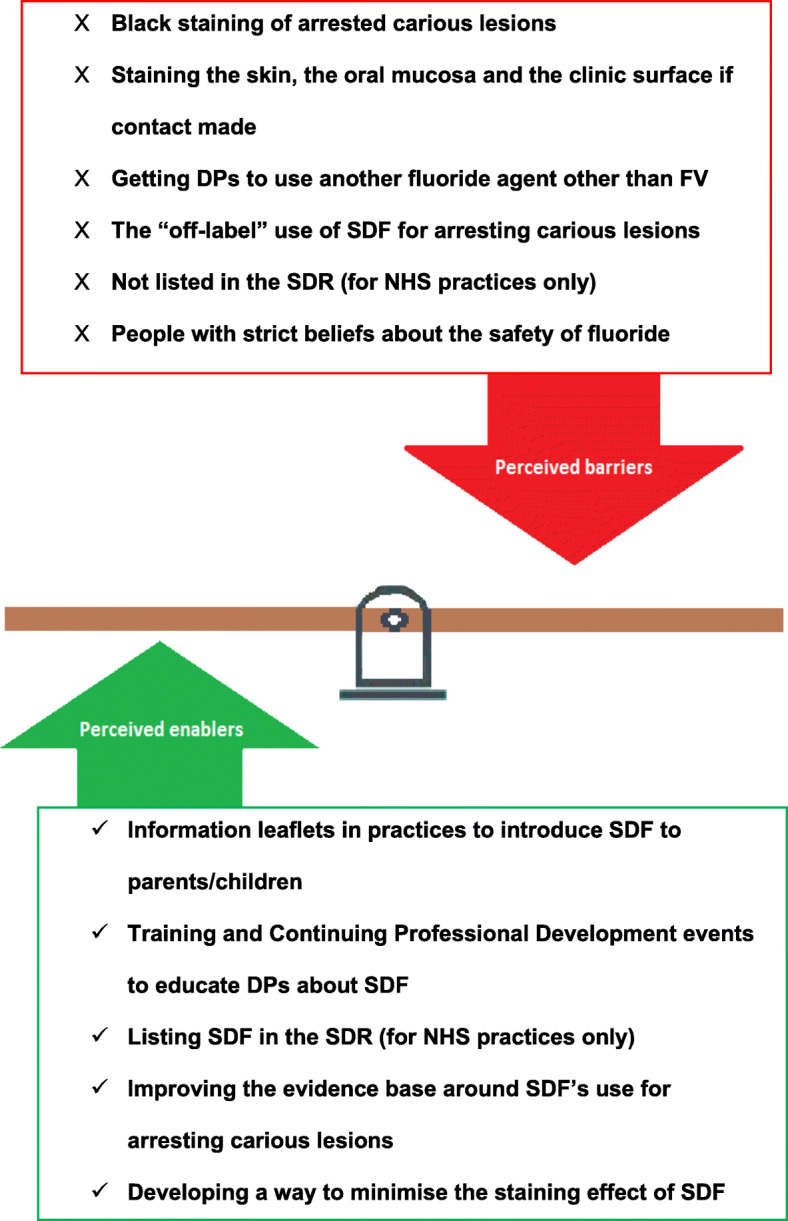
Dental professionals’ perceived barriers and enabler to using SDF in practice

A few participants suggested that improving the evidence base around the use of SDF for arresting carious lesions in children and restricting or minimising the staining effect could increase implementation. It was also suggested that the introduction of SDF into the SDR would allow NHS primary care practitioners to claim for it, hence removing the financial barrier. Figure 1 summarises the perceived barriers and enablers to using SDF in practice.

#### DPs’ perceived uses of SDF

The DPs Interviewed for this study believed that SDF would be a useful option for children unable to cooperate or tolerate other treatment approaches. One dentist went on to say that SDF should be limited to uncooperative children but could also be used for adults with dental anxiety or special needs. The majority of participants agreed that SDF would be particularly beneficial in avoiding or delaying the use of General Anaesthesia (GA) and intimated that parents would rather their child had black teeth, whether in the posterior or anterior sextants, if this avoided their child having a GA. A dental therapist who had experience of applying SDF on a three-years old boy’s anterior and posterior carious lesions commented:

*“Uh, I didn’t really need to convince her (the child’s mother). She was happy to do it if it’s a possibility of avoiding a general anaesthetic”*

**DP 5 (Dental therapist)**.

One dentist identified another potential advantage of SDF was that applying it does not require any complex or advanced equipment, making it particularly useful in developing countries or areas with limited resources. Some DPs also suggested that SDF would be especially valuable where a child has multiple carious lesions, where treating all lesions would normally require several dental appointments. Applying SDF on all carious lesions during one appointment could result in both time and cost savings.

*“you’ve often got the situation where a child has got lots of teeth that need treatment, um, so you could quite easily apply SDF on everything, even at one visit. And that would be a quick, cost-effective way of getting it done”*

**DP 1 (Dentist)**.

#### DPs’ views of SDF compared to HT

Some of those interviewed raised points about the similarities between SDF and the HT. The HT has become increasingly popular in children’s dentistry and has been proven to be effective for managing carious lesions in primary teeth [25]. This was a theme which emerged from the initial few interviews and as a result the interview topic guide was adapted to specifically explore DPs perceptions of the relative advantages and disadvantages of both SDF and the HT. Some DPs interviewed during this study suggested that SDF would be more comfortable for the child due to the simple application process involved, whereas the HT can be uncomfortable when seating the crowns*.*

*“I mean certainly we do use the Hall crown a, a lot and you know, the Hall Technique and that’s, that’s very effective. But even then there are certain things you’ve got to do with it that maybe are slightly uncomfortable you know, putting the separators, actually seating the crowns, um, and they can be quite difficult, quite challenging if the crown, if it’s difficult to match the crown size to the tooth”*

**DP 8 (Dentist)**.

Participants stated that placing the HT requires more cooperation, as fitting the HT crowns has more steps and takes more time than applying SDF.

*“Um, however, I guess the downside of the Hall crown is it does need a little bit more cooperation to do I think than SDF, um, because you need to seal it and remove cement and things like that”*

**DP 9 (Dentist)**.

It was still felt however, that when it comes to SDF, parents may be more sceptical about its effectiveness due to the lesion being left open, and food might keep packing in the area. The area would require careful tooth cleaning to remove the debris. It was suggested that parents may feel more confident about the use of the HT due to the lesion being covered and because it may not require the same level of follow-up care.

*“The only thing about the Hall crown is at least the parent thinks it’s covered so they don’t have to pay so much attention to cleaning they would think in their head, you know, they think oh, it’s covered up whereas they’d be more worried about, “Oh, you’re just putting a paint on and darkening it, you haven’t actually fixed the hole”. So in their head they think why haven’t you fixed the hole?”*

**DP 11 (Dentist)**.

### DPs’ views regarding parents’/children’s acceptability of SDF

Participants had mixed views about how parents may feel about SDF. Some participants believed parents would be reluctant to have SDF used on their children, due to the discolouration of the teeth, while others thought they would not mind the appearance of SDF treated carious lesions.

*“The downside is it does look black so you will get some parents that’ll say, “No, my kid’s not having that done”, I’m sure”*

**DP 1 (Dentist)**.

These beliefs were explored further in the interviews, as were the factors that DPs believed may influence parents’ decision-making. Some participants suggested that fathers may be less concerned about the appearance of their child’s teeth after treatment especially if it was simple and pain-free, whereas mothers may be more concerned about the aesthetics. One participant however, disagreed with this viewpoint:

“*Um, no, I, I don’t think a mother versus father’s opinion would be different”*

**DP 7 (Dentist)**.

Child gender was identified by participants as a potential influence with some participants suggesting that girls are generally more self-conscious than boys. Other participants however, thought that gender would not impact upon the child’s decision-making around SDF. The age of the child at the time of treatment was also identified as potentially influencing parents’ decision-making with some participants suggesting that parents of younger children (six or younger) would be less concerned about discoloration, believing that younger children may not be as self-conscious. It was also perceived that there would be less opposition to SDF being applied to posterior rather than anterior teeth.

When exploring children’s acceptability of SDF, DPs suggested that children generally preferred what they consider to be the least invasive treatment and, therefore, may choose SDF, despite the discolouration. One dentist interviewed suggested that while younger children may be less bothered by the staining, they may also be influenced by their parent’s views.

*“Yeah, younger kids wouldn’t be as self-conscious. They haven’t got the capacity to determine that, unless of course mummy says it’s horrible-looking then they’re probably not going to be very happy with it either”*

**DP 12 (Dentist)**.

Participants believed that older children can be more self-conscious and more accepting of SDF for their posterior teeth, but less so for anterior teeth and may be influenced by their social environment, and therefore may be influenced by other factors, such as the school they attend and the views of their peers.

*“I think it, I think it depends on what environment they’re in, so depending on what school they’re at and the type of school that they’re at, ‘cause children can be cruel”*

**DP 3 (Dental nurse)** *.*

## Discussion

This qualitative study found that DPs’ knowledge and experience of SDF varied significantly, from being unaware of it prior to the interview, to having used it in practice. They saw the main advantages centring on its non-invasive nature and the low levels of patient co-operation required to apply it. The most significant barrier identified was discolouration of the treated tooth and DPs’ concern about parent and child acceptance of this. It was agreed however, that parents and children may be more accepting of SDF and the discolouration associated with it, when treating non-visible lesions or when used in place of more invasive treatments or GA. These findings echo previous survey data on DPs’ perceptions and attitudes toward SDF [18–21], which identified a lack of knowledge about SDF, discolouration as a barrier for parents and highlighted the need for education regarding usage. This seems to be the first qualitative study to explore DPs’ perceptions of SDF use. Qualitative research enables the identification of issues a priori and allows deeper insight into attitudes, not possible through quantitative research [26].

Of the 14 interviewed DPs, 13 had some previous knowledge of SDF. Dental professionals working within a dental school setting, or who had recently graduated, were more informed than those working in general practice for longer. Of the few DPs who had applied SDF, all worked at Dundee Dental Hospital. By working within an educational institution, they may have been more likely to be exposed to novel and innovative treatment approaches. Silver diamine fluoride was licenced in the UK in April 2016 [17] and the interviews were conducted in (December 2018 – March 2019).

The most commonly reported advantage of SDF by the DPs, was that applying it required less compliance on the part of the child, particularly in comparison to other procedures used to manage carious lesions. As a result, SDF was seen as potentially beneficial in acclimatising children to the dental environment, supporting them towards accepting more invasive dental procedures in the future. This is particularly important because traumatic dental experiences, particularly in childhood, have been linked strongly to the development of dental anxiety and dental phobia through adulthood [27].

The most commonly reported disadvantage of SDF, in line with previous studies [28], was the permanent black staining of the arrested carious lesions caused by the formation of silver phosphate [29]. One other perceived disadvantage related to the commercial Riva Star™, SDF product, is that it is a clear solution. This makes accidental spillages difficult to see. Not all SDF products, have this issue, for example Advantage Arrest™ (Elevate Oral Care LLC, West Palm Beach, Florida, USA), marketed in the USA is blue in colour making it easier to spot any inadvertent spillage.

In addition to these clinical level barriers, clinicians believe that parents and children may find the colour change a barrier to accepting the treatment. This has been reported in the literature as a significant disadvantage [20, 28, 30]. The rationale given by DPs was that they thought parents might worry that their child would be bullied at school or nursery because of their physical appearance. Globally, 15.3% of students who have been bullied report being made fun of because of how their face or body looks [31]. They also suggested that parents may be concerned that others would think they have neglected their child’s oral health. DPs considered another potential parental concern would be the potential of accidental contact staining of the gingiva and skin.

Some DPs reported encountering patients who believed that fluoride is harmful despite the assurances of health organisations. There is a small proportion of the population likely to be reluctant to receive any treatment that contains fluoride and their perspective has to be respected. However, raising awareness of the benefits of fluorides in children’s dental health, while addressing people’s concerns, may alleviate some of these concerns [32].

Fluoride varnish is routinely used and national guidance recommends it is applied twice a year for prevention of carious lesions in children aged two and over [33]. Introducing a new fluoride agent and asking clinicians to change well established practices and behaviours may be challenging. Although the indications for SDF and FV overlap but there are some distinct differences. Fluoride varnish is effective at preventing carious lesions [4] whereas SDF’s effectiveness seems to lie more in arresting them [12] and it is more effective than FV in this respect [34]. Recommendations that FV is applied following SDF [35], might promote both materials’ complementary actions [9, 36]. Furthermore, despite evidence of effectiveness and national guidance promoting its use [33], 12 of the 14 NHS Boards in Scotland have not met the UK Government’s HEAT target for FV applications in 2015 [37]. Factors influencing this low uptake remain unclear, however possible explanations are that some DPs are not convinced about the effectiveness of such preventive approaches, or some parents are reluctant to have fluoride therapy for their child.

Some of those interviewed reported that they would be hesitant to use SDF as this would need to be “off-label”. In the UK prescribers can use a product in this way provided they are satisfied that an alternative, licensed medicine would not meet the patient’s requirements and it would serve their needs better, based on the available evidence supporting its efficacy and safety [38]. Although this is common practice in medicine, raising awareness around this for dental prescribers may be required.

To facilitate SDF use, minimising discolouration would seem beneficial. Riva Star™ provide encapsulated potassium iodide KI for application immediately after SDF. However, a recent systematic review [39] reported conflicting evidence and uncertainty for the effectiveness of SDF + KI in mitigating the long-term staining effect of SDF. Even though some studies reported a positive association between SDF + KI and minimal discolouration, other studies refuted these findings while others reported an increased blackening over time.

National clinical guidelines are considered a reliable information source to improve the quality of clinical decision making and assure clinicians about the appropriateness of the treatments they provide [40]. Therefore, incorporation of SDF into clinical guidelines could encourage uptake. This would ideally go hand in hand with remuneration for SDF being incorporated into the SDR as some DPs believe that not being able to claim a fee for its use, is a further practical barrier to using SDF in NHS practices.

Alongside guidance, training events or workshops to familiarise DPs with SDF when it is appropriate to use it as an “off-license” product were suggested by DPs as helpful in overcoming some of their perceived barriers. Having an SDF information sheet in practice was also suggested to help with the introduction of SDF to parents. This would support parents to judge whether the advantages of using SDF outweigh the disadvantages in their individual situation and facilitate decision-making regarding their child’s dental treatment.

Dental professionals believed that SDF may be particularly useful for very young children and those who cannot tolerate other treatments. It could act as a transitional treatment until the child is more able to cooperate. However, it may also be a final treatment if the parents/child do not mind the discolouration. SDF’s ease of use is relevant for older children or adults who cannot tolerate standard treatments for medical or psychological reasons i.e. frail elders or adults with physical disabilities or dental phobias. However, further research to support the effectiveness of SDF in arresting coronal carious lesions in the permanent dentition is needed [41]. This unique benefit of SDF could potentially reduce referrals for GA for some patients. Those living in more in areas with less access to dental care may also benefit as it does require complex equipment for its application. Finally, SDF may also be beneficial where a patient has several lesions that cannot be managed in one. The time lag between appointments in these situations may increase the risk of existing lesions progressing and becoming symptomatic. Applying SDF to all lesions would arrest them and control the disease while awaiting the completion of the treatment.

Interestingly, DPs compared SDF application with placement of crowns using the HT since they share some common clinical indications. Participants believed that SDF would be more convenient for the child than the HT which requires placing elastic separators and scheduling a second visit to fit the crown. Furthermore, seating the crown can result in disruption of the occlusion and the child may experience discomfort after the crown is placed, albeit this resolves within 24 h. Participants also perceived that lower levels of child cooperation would be required when applying SDF but expressed awareness that unexpected movements could result in oral mucosa, skin or clinic surface staining, so a degree of co-operation was still required. Another stated benefit was that SDF could be applied to several carious lesions even on two occluding teeth at the same appointment whereas the HT has some restrictions, for example, it cannot be used on two occluding teeth until the occlusion becomes re-established with bilateral contacts.

For this study, purposive sampling was undertaken to ensure sample diversity. Participants were recruited through primary and secondary care from two NHS Health Boards (HBs) in Scotland. These findings may however be generalisable across the whole UK, considering the similarity in the training DPs receive across UK NHS HBs. It should be noted however, that the primary aim of qualitative research is to gain a greater understanding of opinions and trends and not necessarily identify issues that are generalisable. Furthermore, the remuneration system is not necessarily the same across the four countries of the UK, which in turn may influence the uptake of a specific treatment unless it is listed in all four SDRs in the UK i.e. if SDF is introduced first in the Scottish SDR, it is only logical that a higher uptake of SDF would be in Scotland than the rest of the UK. In order to minimise researcher bias, a proportion of interview transcripts were double coded independently by two authors (one clinical and one non-clinical experienced qualitative researcher).

However, there are a few caveats that should be kept in mind when interpreting the results of this study. Nine out of the 14 DPs who were interviewed had not had experience with SDF before and therefore, their views may change after applying it. In addition, because SDF was only introduced into the UK recently, DPs who had used it before, had limited experience and will not have had the opportunity to follow up patients over extended periods of time. Seeing patients over longer timeframes, allows clinicians to understand long-term outcomes associated with treatment and gain deeper understanding of the impacts on the child and family. It could be the case that with greater experience and follow up with patients, the DPs’ perceptions of the treatment may change. In addition, the interviews were conducted during the working day with busy healthcare professionals and it may be that they condensed their responses or were very focussed in their conversation due to time pressures. However, all participants completed the interview as planned and were asked if there was anything else they wished to add at the end of the interview. As a result, it is unlikely any important contributions were missed.

This study focused on DPs attitudes and acceptability of SDF. These are vital because if DPs are reluctant to use or even learn about SDF, they will not adopt it and parents will not have the opportunity to find out about SDF in the first place. Research exploring the views of parents and children and their acceptability of SDF will complete a fuller picture around the use of SDF in practice.

## Conclusions

Dental professionals were aware that SDF can arrest carious lesions but saw staining of the carious lesion as its major disadvantage and had preconceived ideas that parents would find the appearance a barrier. They considered the application process to be simple, non-invasive and less challenging of child cooperation than other dental procedures, but that a minimum level of compliance would still be required. Dental professionals believed that SDF is a valuable addition to their paediatric dentistry treatment procedures and were prepared to suggest actions that could be taken to reduce each of the barriers they noted.

## Supplementary information

**Additional file 1.** Interview topic guide.

## Data Availability

The datasets used and analysed during the current study and a description of the coding tree are available from the corresponding author on reasonable request.

## Abbreviations

SDF: Silver diamine fluoride
DP: Dental professional
SDPBRN: Scottish Dental Practice Based Research Network
FV: Fluoride varnish
HT: Hall technique
HB: Health Boards
