# Supplementary material for: “It’s really no more difficult than putting on fluoride varnish”: a qualitative exploration of dental professionals’ views of silver diamine fluoride for the management of carious lesions in children
Source: BMC Oral Health. 2020 Sep 15;20:257. doi: 10.1186/s12903-020-01243-y (PMC7490474; doi:10.1186/s12903-020-01243-y)
Supplement: Supplementary file 1 — Additional file 1. Interview topic guide. [file 12903_2020_1243_MOESM1_ESM.docx]

**INTERVIEW TOPIC GUIDE**

***Introduction***

*Firstly, can I just find out a little bit about you ….*

- *What is your role in the practice/clinic?*
- *How long have you been qualified?*
- *How often do you see children in the clinic?*

***Silver Diamine Fluoride***

- *What do you know about SDF?*
- *Have you tried applying SDF? If yes, could you tell me more about it?*
- Are you aware of any colleagues using? Did you provide any feedback?
- *What advantages do you think SDF has over traditional treatment?*
- *What do you think the barriers would be to you using it for carious lesions in primary teeth?*
- *If you had it available to you in practice, what do you think might help you to use it for primary teeth?*
- *For which cases might you consider SDF instead of the current treatments you have available?* Why? What factors would you take into consideration?
